# Polyphenol Intake and Gastric Cancer Risk: Findings from the Stomach Cancer Pooling Project (StoP)

**DOI:** 10.3390/cancers12103064

**Published:** 2020-10-20

**Authors:** Facundo Vitelli-Storelli, Marta Rossi, Claudio Pelucchi, Matteo Rota, Domenico Palli, Monica Ferraroni, Nuno Lunet, Samantha Morais, Lizbeth López-Carrillo, David Georgievich Zaridze, Dmitry Maximovich, María Rubín García, Gemma Castaño-Vinyals, Nuria Aragonés, Manuela Garcia de la Hera, Raúl Ulises Hernández-Ramírez, Eva Negri, Rossella Bonzi, Mary H. Ward, Areti Lagiou, Pagona Lagiou, Malaquías López-Cervantes, Paolo Boffetta, M. Constanza Camargo, Maria Paula Curado, Zuo-Feng Zhang, Jesus Vioque, Carlo La Vecchia, Vicente Martín Sánchez

**Affiliations:** 1 Research Group in Gene-Environment Interactions and Health, Instituto de Biomedicina (IBIOMED), University of León, 24071 León, Spain; fvits@unileon.es (F.V.-S.); mrubig@unileon.es (M.R.G.); vmars@unileon.es (V.M.S.); 2 Department of Clinical Sciences and Community Health, University of Milan, 20122 Milan, Italy; marta.rossi@unimi.it (M.R.); claudio.pelucchi@unimi.it (C.P.); monica.ferraroni@unimi.it (M.F.); rossella.bonzi@unimi.it (R.B.); carlo.lavecchia@unimi.it (C.L.V.); 3 Department of Molecular and Translational Medicine, University of Brescia, 25121 Brescia, Italy; matteo.rota@unibs.it; 4 Cancer Risk Factors and Life-Style Epidemiology Unit, Institute for Cancer Research, Prevention and Clinical Network, ISPRO, 08518 Florence, Italy; d.palli@ispro.toscana.it; 5 Department of Epidemiology, EPIUnit–Instituto de Saúde Pública da Universidade do Porto, 4050-091 Porto, Portugal; nlunet@med.up.pt (N.L.); samantha.morais@ispup.up.pt (S.M.); 6 Departamento de Ciências da Saúde Pública e Forenses e Educação Médica, Faculdade de Medicina da Universidade do Porto, 4200-319 Porto, Portugal; 7 Mexico National Institute of Public Health, Morelos 62100, Mexico; lizbeth@insp.mx; 8 Department of Epidemiology and Prevention, Russian N.N. Blokhin Cancer Research Center, 115478 Moscow, Russia; dgzaridze@crc.umos.ru (D.G.Z.); dmax@crc.umos.ru (D.M.); 9 Consortium for Biomedical Research in Epidemiology and Public Health (CIBERESP), 28029 Madrid, Spain; gemma.castano@isglobal.org (G.C.-V.); nuria.aragones@salud.madrid.org (N.A.); manoli@umh.es (M.G.d.l.H.); 10 IMIM (Hospital del Mar Medical Research Institute), 08003 Barcelona, Spain; 11 Department of Public health, Universitat Pompeu Fabra (UPF), 08002 Barcelona, Spain; 12 Barcelona Institute for Global Health—ISGlobal, 08036 Barcelona, Spain; 13 Cancer Epidemiology Section, Public Health Division, Department of Health of Madrid, 28035 Madrid, Spain; 14 Department of Public Health, Instituto de Investigación Sanitaria y Biomédica de Alicante, ISABIAL-UMH, 46020 Alicante, Spain; 15 Department of Biostatistics, Yale School of Public Health, New Haven, CT 06520, USA; raul.hernandezramirez@yale.edu; 16 Department of Biomedical and Clinical Sciences, University of Milan, 20157 Milan, Italy; eva.negri@unimi.it; 17 Division of Cancer Epidemiology and Genetics, National Cancer Institute, Rockville, MD 20850, USA; wardm@exchange.nih.gov (M.H.W.); maria.camargo@nih.gov (M.C.C.); 18 Department of Public and Community Health, School of Health Sciences, University of West Attica, 11521 Athens, Greece; alagiou@uniwa.gr; 19 Department of Hygiene, Epidemiology and Medical Statistics, School of Medicine, National and Kapodistrian University of Athens, 10561 Athens, Greece; plagiou@hsph.harvard.edu; 20 Department of Epidemiology, Harvard T.H. Chan School of Public Health, Boston, MA 02115, USA; 21 Department of Public Health, Facultad de Medicina, UNAM, Coyoacán 04810, Mexico; mlopezcervantes@unam.mx; 22 Stony Brook Cancer Center, Stony Brook University, Stony Brook, NY 11794, USA; paolo.boffetta@mssm.edu; 23 Department of Medical and Surgical Sciences, University of Bologna, 40126 Bologna, Italy; 24 Centro Internacional de Pesquisa, A. C. Camargo Cancer Center, Sao Paulo 01508-010, Brazil; mp.curado@cipe.accamargo.org.br; 25 Department of Epidemiology, UCLA Fielding School of Public Health and Jonsson, Comprehensive Cancer Center, Los Angeles, CA 90095-6900, USA; zfzhang@ucla.edu

**Keywords:** diet, epidemiology, flavonoids, gastric cancer, polyphenols

## Abstract

**Simple Summary:**

Gastric cancer (GC) has the fifth highest incidence of any cancer type worldwide and the third highest mortality rate, so its prevention is very important. Among dietary factors, the consumption of fruit and vegetables has been inversely related to GC risk. Phenolic compounds may exert a favorable effect on the risk of several cancer types, including gastric cancer. However, selected polyphenol classes have not been adequately investigated in relation to GC. There is, however, no comprehensive analysis of polyphenols and GC risk methods to date. In order to provide a detailed evaluation of the relationship between dietary intake of polyphenols and GC risk, we analyzed data from the Stomach cancer Pooling (StoP) Project consortium.

**Abstract:**

Phenolic compounds may exert a favorable effect on the risk of several cancer types, including gastric cancer (GC). However, selected polyphenol classes have not been adequately investigated in relation to GC. The aim of this study is to evaluate the association between the intake of polyphenols in relation to GC risk. We used data from the Stomach cancer Pooling (StoP) Project, including 10 studies from six countries (3471 GC cases and 8344 controls). We carried out an individual participant data pooled analysis using a two-stage approach. The summary odds ratios (ORs) of GC for each compound, and the corresponding 95% confidence intervals (95% CI), were computed by pooling study specific ORs obtained through multivariate logistic regression, using random effect models. Inverse associations with GC emerged for total polyphenols (OR = 0.67, 95% CI = 0.54–0.81, for the highest versus lowest quartile of intake), total flavonoids (OR = 0.73, 95% CI = 0.55–0.90), anthocyanidins (OR = 0.74, 95% CI = 0.56–0.92), flavanols (OR = 0.77, 95% CI = 0.66–0.88), flavanones (OR = 0.57, 95%CI = 0.44–0.69), total phenolic acids (OR = 0.75, 95%CI = 0.55–0.94), and hydroxybenzoic acids (OR = 0.73, 95%CI = 0.57–0.89). Results were consistent across strata of age, sex, social class, and smoking habit. Suggestive inverse associations were also found for flavonols (OR = 0.76, 95%CI = 0.51–1.01) and hydroxycinnamic acids (OR = 0.82, 95%CI = 0.58–1.06). Further investigations from longitudinal data are needed to confirm this association.

## 1. Introduction

The incidence rates of gastric cancer (GC) show a wide geographical variability, which has been attributed to different exposures, including *Helicobacter pylori* infection, smoking, diet, and nutrition, besides genetic factors [1,2,3]. Among dietary factors, the consumption of fruit and vegetables has been inversely related to GC risk [4,5,6]. This inverse relationship has been attributed to the intake of nutrients, micronutrients, and other food compounds, including polyphenols [7].

Polyphenols are secondary phenolic metabolites present in a wide variety of vegetables fruits, cereals, dry legumes, chocolate, and spices. They can be classified into five classes, flavonoids, phenolic acids, stilbenes, lignans, and other polyphenols. Most studies have focused on the relation between flavonoids and GC, with inconclusive results [8,9,10]. A case–control study from Greece [9] found that flavanone intake was inversely related to GC. Another case–control study, from Spain, reported an inverse association for the intakes of flavonoids, anthocyanidins, and flavanols [10]. A meta-analysis based on six studies, three case–control and three cohort studies from Europe, Asia, and USA, showed that dietary total flavonoids intake was inversely associated with the risk of GC in the European population, but not in US or Asia [11]. As far as we know only one study in Mexico has analyzed the effect of total polyphenol intake and a few classes of polyphenols in relation to GC risk, showing a higher risk of GC among those with a low total polyphenol intake and a high intake of nitrites, and an approximately double risk among those with lower intakes of cinnamic acids, secoisolariciresinol, and coumestrol [12]. However, a comprehensive analysis of the effect of total polyphenol intake and all the specific classes of polyphenol on GC risk is still lacking. In order to provide a detailed evaluation of the relationship between dietary intake of polyphenols and GC risk, we analyzed data from the Stomach cancer Pooling (StoP) Project consortium [13].

## 2. Materials and Methods

### 2.1. Participant Studies, Data Collection, and Harmonization

The StoP Project is a consortium of epidemiological studies on GC (www.stop-project.org). Details of the aims and methods of the consortium have been provided elsewhere [13]. Briefly, data for the current analysis were based on the second release of the combined database, including 31 GC studies worldwide. Data sets of the original studies were centrally collected, validated [14], and harmonized according to a specified format [13]. For these analyses, 10 studies from six countries, including a total of 3471 cases and 8344 controls, were selected based on availability of data to calculate polyphenols consumption (Table 1). Two studies were from Italy [15,16], one from Greece [17], one from Russia [18], one from Portugal [19], two from Spain [20,21], and three from Mexico [12,22,23]. Investigators who agreed to participate signed a Data Transfer Agreement and, transferred a copy of the complete original data set of the study. The University of Milan Institutional Review Board provided the ethical approval for the StoP Project (reference 19/15—01 April 2015).

### 2.2. Analysis of Polyphenols Intake

All the included studies assessed the participants’ dietary habits using their own specific food frequency questionnaires (FFQ) focusing on usual diet before diagnosis (for cases), onset of disease or hospital admission (for hospital-based controls), or recruitment (for population-based controls). The FFQ foods that were included in the assessment of polyphenol intake included vegetables and legumes, fruits, sweets, cereals, alcohol, juices, and other beverages. The dietary intake of polyphenols was estimated using the Phenol-Explorer (http://phenol-explorer.eu/) and expressed as aglycone from both chromatography and chromatography after hydrolysis analytic methods [24]. We used aglycone equivalents in order to standardize data from the results of different analytical methods and to facilitate cross-study comparisons [25]. The estimation of polyphenols content in foods included in the Phenol-Explorer expressed as aglycones were calculated through the following equation (Equation (1)).

(1) $$P\;aglycone_{\frac{mg}{100\;mg}}=\left(\frac{\left[glucoside\right]\;}{Pm\;glucoside}\right)\times Pm\;aglycone$$

These values were used to compute the polyphenols intakes in milligrams per day from the FFQ information per each subject. No retention factors were applied in the calculation of the amount of polyphenols ingested.

### 2.3. Analysis of Polyphenols Intake

We carried out an individual participant data pooled analysis using a two-stage approach [26]. First, we assessed the relationship between polyphenols intake and GC by computing, for each study, the odds ratios (ORs) and the corresponding 95% confidence intervals (CIs) using multivariable unconditional logistic regression models. The models used in this study were adjusted for age, social class (low, intermediate, high), GC family history, body mass index, sex, smoking status (never, former, current low, current intermediate, current high), consumption of salt, and alcohol intake (never, ≤12, >12 ≤ 47, >47 g/day). Furthermore, phenolic compounds intake was adjusted for total energy using the residuals method [27], and posteriorly categorized in quartiles according to its distribution among controls. In the second stage, summary (pooled) ORs and the corresponding 95% CIs were estimated using a random-effect model [28]. Heterogeneity between studies was assessed by using the Q test and I^2^ statistic. Moreover, a number of stratified analyses were performed, according to sex, age group (≤60, >60 years), social class (low, intermediate or high), and smoking status (never smokers, ever smokers). Stata software release 14 [29] was used for mixed effects logistic regression, as well as Python version 3.14 [30] and R version 3.6 [31] for the extraction of Phenol Explorer data on polyphenol content in aliment, and for the estimation of the polyphenol intake by the individuals, respectively.

## 3. Results

The pooled ORs and the corresponding 95% CI for total polyphenols, total flavonoids and total phenolic acids according to their quartiles of intake, as well as using continuous variables of intake, are shown in Table 2.

For most polyphenols, the ORs were lower in the highest quartile intake, as compared to preceding ones, though in the absence of a linear trend. The ORs for the highest vs. the lowest quartile were 0.67 (95% CI = 0.54–0.81) for total polyphenols, 0.75 (95% CI = 0.55–0.94) for total phenolic acids, and 0.73 (95% CI = 0.55–0.90) for total flavonoids. Regarding flavonoid subclasses, the ORs for highest vs. the lowest quartile were 0.74 (95% CI = 0.56–0.92) for anthocyanidins, (0.77, 95% CI = 0.66–0.88) for flavanols, and 0.57 (95% CI = 0.44–0.69) for flavanones. With reference to total phenolic acids classes, the OR was 0.73 (95% CI = 0.57–0.89) for hydroxybenzoic acids. The ORs were 0.76 (95% CI = 0.51–1.01) for flavonols and 0.82 (95%CI = 0.58–1.06) for hydroxycinnamic acids. Continuous ORs, computed for an increase of 1 standard deviation of each polyphenol, ranged between 0.84 (95% CI = 0.78–0.90) for flavanones and 0.93 for anthocyanidins (95% CI = 0.86–1.01), flavanols (95% CI = 0.85–1.00), total phenolic acids (95% CI = 0.81–1.04), and hydroxycinnamic acids (95% CI = 0.82–1.05).

**Figure 1:**
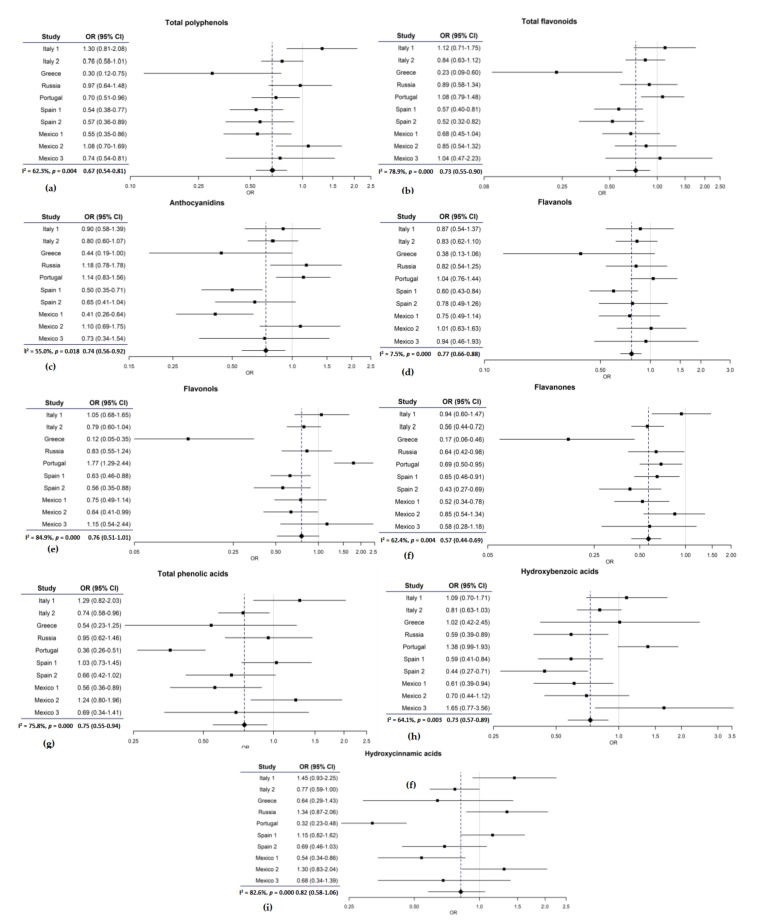
Study specific and pooled odds ratios (OR) and their 95% confidence intervals (CI) for quartile 4 vs. quartile 1 of polyphenol intake; (**a**) pooled analysis of polyphenols; (**b**) pooled analysis of flavonoids; (**c**) pooled analysis of anthocyanidins; (**d**) pooled analysis of flavanols; (**e**) pooled analysis of flavonols; (**f**) pooled analysis of flavanones; (**g**) pooled analysis of phenolic acids; (**h**) pooled analysis of hydroxybenzoic acids; (**i**) pooled analysis of hydroxycinnamic acids.

**Figure 2:**
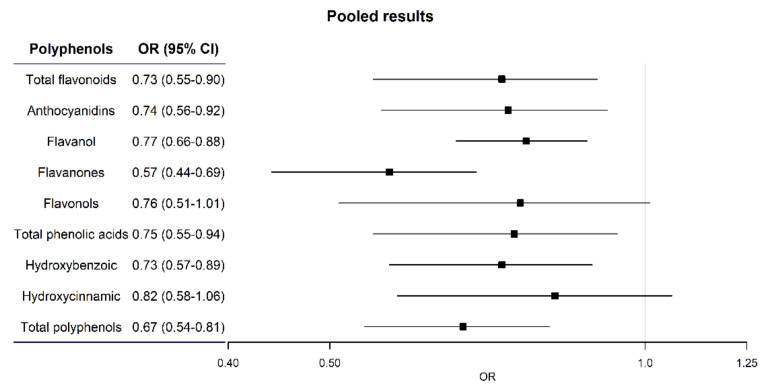
Pooled OR and 95% CI of selected polyphenols intake.

Figure 1 presents the study specific ORs for the highest vs. lowest quartile of polyphenols in each study. Despite appreciable heterogeneity, most study specific ORs were below 1. The pooled ORs for the highest vs. the lowest quartile of each polyphenol are also summarized in Figure 2.

Table 3 gives the ORs for the highest vs. the lowest quartile of intake of polyphenols in strata of sex, age, social class indicators, and smoking status. All the inverse associations were consistent across strata of the covariates considered.

## 4. Discussion

This uniquely large collaborative pooled analysis of original data indicates an inverse association between the intake of total polyphenols, total flavonoids, and total phenolic acids and GC risk. Among flavonoids, we found a reduced risk of GC associated with higher intake of anthocyanidins, flavanols, and flavanones; among phenolic acids, hydroxybenzoic acids intake was inversely related to GC risk.

Our collaborative reanalysis is therefore consistent with some, though not all, previous evidence. However, no study investigated the association between total polyphenols and the GC risk to date. With reference to flavonoids, an inverse association between the total intake of their subclasses and GC risk was found in a case–control study from Spain including 354 cases [32]. Furthermore, a meta-analysis on total flavonoids and digestive tract cancers found that flavonoids were inversely related to the risk of GC in the European population but not in the US or in Asia [11]. Prospective studies, two from Finland including 111 and 74 GC cases [33,34], and two from the USA including 248 and 1297 cases [7,35], did not find any association with total flavonoids. However, the European Prospective Investigation into Cancer (EPIC) [36] study found an inverse association for total flavonoids among women, but not among men.

With reference to flavonoid subclasses, a number of studies indicated an inverse relation between the intake of flavanol and GC, in line with our results [37,38,39]. A case–control study from Italy, including 230 GC cases, reported an inverse association between proanthocyanidins, a subclass of flavanols, and GC risk [37]. In a case–control study from Korea, including 334 GC cases, inverse associations were observed for flavan-3-ols, which is part of flavanols, besides total dietary flavonoids [38]. In another case–control study conducted in Spain on 329 GC cases, an inverse relationship for the intake of flavan-3-ols, as well as total flavonoids (estimated as aglycones) was found [10]. A meta-analysis of epidemiologic studies on flavan-3-ols and cancer risk also revealed an inverse association with GC risk among women, but not men [39].

With reference to anthocyanidins, the already cited EPIC study, including 683 incident GC cases, reported an inverse association with GC risk in women [36], but a subsequent meta-analysis on anthocyanidins and GC risk reveled no association [40].

A case–control study from Greece including 110 cases of GC found inverse associations with flavanones and flavonols [9]. Another Swedish population-based case–control study including 505 GC cases reported an inverse association with the flavonol quercetin [41]. Likewise, a meta-analysis involving 4593 cases supported the evidence of an inverse association between flavonols and GC risk [42].

With reference to phenolic acids, only one case–control study from Mexico on 248 cases investigated them in relation to the GC risk [12], suggesting an inverse association with cinnamic acid, a derivate from hydroxycinnamic acids.

Our pooled study is the first to consider the association between hydroxybenzoic acids and total polyphenols and GC risk. In addition, the present study is the largest to date on polyphenols and GC and has therefore adequate power to quantify the associations considered. For the purpose of this work, a database was generated including all available information on polyphenols content from Phenol-Explorer, together with the mixing of data extracted from chromatography after hydrolysis and chromatography and the food ponderations from FFQ. Aglycones can be absorbed from the small intestine, but most polyphenols are present in the form of glycosides, polymers or esters, that cannot be absorbed in native form [43], needing a transformation by microbiota to aglycones to be absorbed. Thus, estimating polyphenols as aglycones is another strength of this investigation, as this allowed to obtain a more accurate approximation to real consumption of the bioactive structures [44], to reduce the overestimation of the polyphenols and to take advantage of the most complete food composition tables.

A limitation of our collaborative re-analysis is related to the variable accuracy of each study FFQ, with different levels of details and completeness of information. Moreover, the total polyphenols intake was calculated including all classes of polyphenols with the exception of lignans. In addition, the aglycone polyphenols content in food was estimated without taking into account the retention factors. However, this data was not available for most individual polyphenols. Furthermore, the metabolization of polyphenols occurs after ingestion, both at hepatic and colonic level [45,46], and varies among individuals. Part of the variability between the results from studies can be attributed to the heterogeneity of dietary pattern of each country [47], since the polyphenols content in foods varies according to the different factors related with the climate stress, geography, and storage conditions [45,48]. For example, culinary preparation strongly influences the polyphenol availability of foods, quercetin can be reduced by up to 80% from boiling [48]. More detailed information on the main sources and availability of polyphenol classes can be found elsewhere [49]. A decreased risk of GC was found for all polyphenols, with a levelling in the ORs from the second quartile of intake onwards. The type of inverse association observed, therefore, tended to indicate an excess risk of GC for subjects with lowest (inadequate) intake.

The inverse associations observed may partially be due to an effect of a diet rich in fruit and vegetable on GC risk [4,19,50], rather than being specifically attributable to polyphenols intake. Fruits and vegetables are a good source of polyphenols but also for other antioxidant nutrients such as vitamin C which have been also related to a lower risk of GC. Interactions between polyphenol and other nutrients cannot be ruled out and should be explored in more depth in further studies. In particular, there is evidence of an inverse association of citrus fruits [51], which are the major food sources of flavanones. Additionally, consumption of tea, an important contributor of flavanols and hydroxybenzoic acids, could partially explain the inverse association, since numerous studies reported a protective effect of this beverage on GC risk [52].

Polyphenols have several anticarcinogenic properties including antioxidant [44,53] and anti-inflammatory effects [54,55,56], and some of them have shown antimicrobial effects, inhibiting the growth of *Helicobacter pylori* [57,58], a risk factor for GC. In addition, flavonoids interact with a wide range of molecules involved in apoptosis and cell proliferation pathways by affecting their expression or activity [59]. In particular, epigallocatechin-3-gallate, a compound of flavanols, can induce apoptosis of GC cells lines [60]. Naringenin, a compound of flavanones, inhibits the cancer cells proliferation and migration and induces apoptosis in GC [61]; anthocyanidins can induce autophagy and apoptosis on human GC cells [62]; gallic acid, a hydroxybenzoic acid, can act as a metastasis inhibitor of GC specific cell lines [63].

## 5. Conclusions

In conclusion, our study suggests that the higher intake of polyphenols (total and specific types) may be associated with a lower GC risk. The mechanisms for a protective effect of polyphenols could be related to their antioxidant, antimicrobial, and pro-apoptotic effects. The relation of dietary polyphenols and GC risk should be further investigated in longitudinal studies.

## Figures and Tables

**Table 1:** Distribution of 3471 gastric cancer cases and 8344 controls of each study included in the StoP (Stomach cancer Pooling) Project (version 2.0).

StoP Project					
Study	Study Type	Country ^1^	Cases (%)	Control (%)	Total ^2^ (%)
La Vecchia et al. [15]	Hospital-based	Italy 1	223 (29.2)	541 (70.8)	764 (6.4)
Ferraroni et al. [16]	Population-based	Italy 2	1001 (46.6)	1145 (53.3)	2146 (18)
Trichipoulos et al. [9]	Hospital-based	Greece	105 (51.7)	98 (48.2)	203 (1.7)
Zaridze et al. [18]	Hospital-based	Russia	361 (43.2)	474 (56.8)	835 (7)
Lunet et al. [19]	Population-based	Portugal	577 (26.8)	1578 (73.2)	2155 (18.0)
Aragonés;Martín et al. [20]	Population-based	Spain 1	329 (10.9)	2700 (89.1)	3029 (25.3)
Vioque et al. [21]	Hospital-based	Spain 2	317 (44.5)	396 (55.5)	713 (7.2)
Lopez-Carrillo et al. [12]	Population-based	Mexico 1	248 (34.2)	478 (65.8)	726 (6)
Lopez-Carrillo et al. [22]	Population-based	Mexico 2	220 (22.6)	752 (77.4)	972 (8.1)
Lopez-Carrillo et al. [23]	Hospital-based	Mexico 3	90 (33.3)	182 (66.7)	272 (2.3)
Total	–	–	3471 (100)	8344 (100)	11,815 (100)

^1^ Number of studies from each country. ^2^ Number of cases and controls of each study.

**Table 2:** Distribution of 3471 gastric cancer cases and 8344 controls according to quartile of intake of selected polyphenols and corresponding odds ratio (ORs) and 95% confidence intervals (CI).

Polyphenol Class	Variables	Quartile of Polyphenols Intake ^b^				OR Continuous ^c^
		1 ^d^	2	3	4	
Total polyphenols	Mean intake (mg/day) ^a^	285.6	402.6	534.2	853.0	0.88 (0.78–0.97)
	Cases	991	809	846	825	
	OR (95% CI)	1	0.73 (0.59–0.88)	0.72 (0.54–0.89)	0.67 (0.54–0.81)	
Total flavonoids	Mean intake (mg/day) ^a^	123.7	184.1	256.4	415.0	0.90 (0.82–0.98)
	Cases	942	814	830	885	
	OR (95% CI)	1	0.78 (0.67–0.88)	0.76 (0.58–0.94)	0.73 (0.55–0.90)	
Anthocyanidins	Mean intake (mg/day) ^a^	12.8	21.9	36.9	79.3	0.93 (0.86–1.01)
	Cases	951	779	857	884	
	OR (95% CI)	1	0.65 (0.52–0.85)	0.78 (0.62–0.93)	0.74 (0.56–0.92)	
Flavanols	Mean intake (mg/day) ^a^	33.9	54.0	90.6	179.6	0.93 (0.85–1.00)
	Cases	903	841	837	891	
	OR (95% CI)	1	0.84 (0.73–0.95)	0.73 (0.67–0.88)	0.77 (0.66–0.88)	
Flavonols	Mean intake (mg/day) ^a^	19.6	30.2	36.7	61.2	0.90 (0.80–1.00)
	Cases	982	818	798	873	
	OR (95% CI)	1	0.75(0.62–0.88)	0.70 (0.49–0.91)	0.76 (0.51–1.01)	
Flavanones	Mean intake (mg/day) ^a^	7.1	19.9	34.8	59.0	0.84 (0.78–0.90)
	Cases	1173	934	716	648	
	OR (95% CI)	1	0.73 (0.55–0.92)	0.60 (0.40–0.79)	0.57 (0.44–0.69)	
Total phenolic acids	Mean intake (mg/day) ^a^	126.5	191.2	269.9	474.5	0.93 (0.81–1.04)
	Cases	1023	843	822	783	
	OR (95% CI)	1	0.76 (0.60–0.92)	0.78 (0.68–0.88)	0.75 (0.55–0.94)	
Hydroxybenzoic acids	Mean intake (mg/day) ^a^	16.3	20.4	27.6	64.3	0.91 (0.85–0.98)
	Cases	959	875	813	824	
	OR (95% CI)	1	0.87 (0.73–1.02)	0.73 (0.55–0.91)	0.73 (0.57–0.89)	
Hydroxycinnamic acids	Mean intake (mg/day) ^a^	102.7	163.3	239.0	425.0	0.93 (0.82–1.05)
	Cases	1004	866	794	807	
	OR (95% CI)	1	0.81 (0.63–0.99)	0.75 (0.65–0.85)	0.82 (0.58–1.06)	

^a^ Computed according the distributions among controls. ^b^ Estimated by two-stage meta-analysis using unconditional logistic regression models adjusted for age, sex, social class, alcohol consumption, body mass index, family history of gastric cancer, smoking status, consumption of salt, and according to the residual model. ^c^ Estimated for an increment of intake equal to 1 standard deviation (computed according the distributions among controls). ^d^ Reference category.

**Table 3:** Pooled odds ratio (OR) and corresponding 95% confidence interval (CI) of gastric cancer for the highest compared to the lowest study-specific quartile of the distribution of polyphenols classes intake, according to strata of selected variables.

Variable	OR (CI 95%) ^a^							
	Sex		Age		Social Class		Smoking Status	
	Men	Women	≤60 years	>60 years	Low	Medium-High	Never Smoker	Smoker
Total polyphenols	0.61 (0.44–0.78)	0.67 (0.52–0.83)	0.71 (0.56–0.87)	0.60 (0.43–0.77)	0.59 (0.46–0.73)	0.71 (0.54–0.88)	0.64 (0.48–0.80)	0.62 (0.45–0.78)
Total flavonoids	0.65 (0.45–0.86)	0.74 (0.56–0.92)	0.67 (0.49–0.85)	0.74 (0.51–0.96)	0.65 (0.42–0.88)	0.73 (0.58–0.89)	0.65 (0.48–0.83)	0.63 (0.44–0.82)
Anthocyanidins	0.68 (0.46–0.90)	0.79 (0.60–0.98)	0.66 (0.52–0.81)	0.78 (0.56–1.00)	0.63 (0.44–0.81)	0.74 (0.56–0.93)	0.78 (0.56–1.00)	0.64 (0.47–0.81)
Flavanols	0.76 (0.59–0.92)	0.72 (0.54–0.90)	0.80 (0.62–0.97)	0.70 (0.53–0.88)	0.72 (0.55–0.89)	0.77 (0.61–0.93)	0.65 (0.49–0.80)	0.78 (0.63–0.93)
Flavonols	0.66 (0.43–0.89)	0.75 (0.48–1.01)	0.71 (0.55–0.87)	0.74 (0.43–1.04)	0.77 (0.44–1.11)	0.66 (0.53–0.79)	0.73 (0.57–0.89)	0.67 (0.52–0.82)
Flavanones	0.55 (0.39–0.71)	0.56 (0.43–0.68)	0.48 (0.35–0.61)	0.59 (0.43–0.74)	0.51 (0.31–0.71)	0.61 (0.48–0.73)	0.56 (0.42–0.70)	0.52 (0.37–0.67)
Total phenolic acids	0.66 (0.46–0.86)	0.65 (0.45–0.84)	0.78 (0.50–1.06)	0.62 (0.43–0.81)	0.55 (0.39–0.71)	0.74 (0.50–0.97)	0.68 (0.46–0.91	0.67 (0.50–0.84)
Hydroxybenzoic acids	0.65 (0.49–0.80)	0.81 (0.48–1.14)	0.72 (0.53–0.92)	0.59 (0.40–0.78)	0.77 (0.53–1.00)	0.62 (0.39–0.84)	0.77 (0.48–1.06)	0.67 (0.55–0.80)
Hydroxycinnamic acids	0.74 (0.48–1.00)	0.67 (0.45–0.90)	0.87 (0.55–1.18)	0.69 (0.45–0.93)	0.56 (0.35–0.76)	0.86 (0.56–1.16)	0.69 (0.44–0.93)	0.75 (0.52–0.99)

^a^ Estimated by two-stage meta-analysis using unconditional logistic regression models including terms for age, sex, social class, alcohol consumption, body mass index, family history of gastric cancer, smoking status, consumption of salt, and energy residuals model.
